# Incidence of Respiratory Syncytial Virus Infection in Older Adults: Limitations of Current Data

**DOI:** 10.1007/s40121-023-00802-4

**Published:** 2023-05-23

**Authors:** Mark H. Rozenbaum, Elizabeth Begier, Samantha K. Kurosky, Jo Whelan, Danai Bem, Koen B. Pouwels, Maarten Postma, Louis Bont

**Affiliations:** 1 Pfizer Inc., Capelle a/d IJssel, The Netherlands; 2 Pfizer Vaccines, Dublin, Ireland; 3 Pfizer Inc., New York, NY USA; 4 HEOR Ltd, Cardiff, UK; 5 University of Oxford, Oxford, UK; 6 University of Groningen, Groningen, The Netherlands; 7 University Medical Center Utrecht, Utrecht, The Netherlands

**Keywords:** Epidemiology, Health policy, Respiratory syncytial virus, Vaccination

## Abstract

**Introduction:**

Respiratory syncytial virus (RSV) is an important cause of severe respiratory illness in older adults and adults with respiratory or cardiovascular comorbidities. Published estimates of its incidence and prevalence in adult groups vary widely. This article reviews the potential limitations affecting RSV epidemiology studies and suggests points to consider when evaluating or designing them.

**Methods:**

Studies reporting the incidence or prevalence of RSV infection in adults in high-income Western countries from 2000 onwards were identified via a rapid literature review. Author-reported limitations were recorded, together with presence of other potential limitations. Data were synthesized narratively, with a focus on factors affecting incidence estimates for symptomatic infection in older adults.

**Results:**

A total of 71 studies met the inclusion criteria, most in populations with medically attended acute respiratory illness (ARI). Only a minority used case definitions and sampling periods tailored specifically to RSV; many used influenza-based or other criteria that are likely to result in RSV cases being missed. The great majority relied solely on polymerase chain reaction (PCR) testing of upper respiratory tract samples, which is likely to miss RSV cases compared with dual site sampling and/or addition of serology. Other common limitations were studying a single season, which has potential for bias due to seasonal variability; failure to stratify results by age, which underestimates the burden of severe disease in older adults; limited generalizability beyond a limited study setting; and absence of measures of uncertainty in the reporting of results.

**Conclusions:**

A significant proportion of studies are likely to underestimate the incidence of RSV infection in older adults, although the effect size is unclear and there is also potential for overestimation. Well-designed studies, together with increased testing for RSV in patients with ARI in clinical practice, are required to accurately capture both the burden of RSV and the potential public health impact of vaccines.

**Supplementary Information:**

The online version contains supplementary material available at 10.1007/s40121-023-00802-4.

## Key Summary Points

**Table:** 

RSV is recognized as a cause of severe respiratory illness in older and at-risk adults, but reported incidence and prevalence rates vary widely between studies.
Our review of the published literature assessing RSV epidemiology in older adults identified lack of tailoring of the sampling period, failure to stratify results by age, limited sample size and absence of measures of uncertainty, as commonly reported limitations across studies.
Due to these limitations, a considerable proportion of studies are likely to underestimate the incidence of RSV infection in older adults; with several RSV vaccines currently under development and likely available already in 2023, this has the potential to underestimate the health benefit that can be gained from a vaccination program in older adults with RSV.
Well-designed studies and increased testing for RSV in patients with ARI are required to capture the clinical and economic burden of RSV and adequately inform vaccine policy in older adults.

## Introduction

Respiratory syncytial virus (RSV), well characterized in infants, is now recognized as an important cause of severe respiratory illness in older adults and adults with respiratory or cardiovascular comorbidities or severe immunosuppression [1–3]. RSV infection and reinfection occur throughout life and are generally mild in healthy younger adults but can cause serious complications in the older adult population [1, 3, 4]. Symptoms in older adults are variable and non-specific, ranging from a mild cold-like illness to respiratory failure [1], and can require hospitalization due to the onset of bronchiolitis and pneumonia in some cases [5, 6]. As RSV cannot be clinically distinguished from other respiratory viruses, laboratory diagnosis is required on the basis of viral detection [by polymerase chain reaction (PCR)] or serology (the detection of antibodies) [1, 7].

Morbidity in older adults can be severe—a comparison of outcomes between hospitalized adults aged ≥ 60 years testing positive for either RSV or influenza found that RSV was associated with increased odds for longer length of stay, pneumonia, intensive care unit admission, exacerbation of chronic obstructive pulmonary disease, and death within 1 year of admission [8]. RSV was estimated to be responsible for approximately 214,000 (95% CI 100,000–459,000) acute lower respiratory infection hospitalizations per year in adults aged ≥ 65 in industrialized countries [9], while another study in the same age group estimated 177,525 RSV-related hospitalizations per year in the USA alone [10]. Mortality is strongly related to the presence of comorbidities or presence in a long-term care facility [10], but estimates of case fatality rates vary [9, 11, 12].

Substantially fewer RSV incidence publications are available for adults than for infants and children, with wide variation in the reported rates. Estimates are often in the form of prevalence studies, reporting the proportion of a sample of individuals with respiratory illnesses who test positive for RSV; this has been termed the ‘incidence proportion’ by some authors [11]. Applying such proportions to numbers of patients with various clinical diagnoses associated with RSV infection has been used to estimate incidence or number of hospitalizations [6, 10]. In addition, several reviews and meta-analyses have attempted to synthesize the literature to provide broader estimates of RSV incidence rates. However, these estimates are subject to the methodological limitations of the original studies [9, 11, 13], which may potentially result in a significant bias in incidence estimates of RSV [9, 13].

Several adult RSV vaccines are currently in development, with licensing in older adults likely in 2023. Accurate estimation of the incidence of RSV in this group is important to fully understand its clinical and economic burden so that informed policy decisions can be made around the implementations of vaccines once approved. This paper reviews the limitations of RSV incidence and prevalence studies in adults with a focus on older age groups in selected high-income countries (the setting where vaccines are initially expected to be taken up), and qualitatively assesses the potential implications for vaccine-related policy decisions. It also suggests points to consider when evaluating or designing epidemiological studies of RSV.

## Methods

A rapid literature review was undertaken to summarize limitations in studies assessing the incidence and prevalence of RSV in adults in high-income Western health systems and examine the potential impact on current RSV epidemiologic estimates. Identified limitations were categorized as author-reported limitations or other study limitations. Author-reported limitations were defined as study limitations explicitly stated by the authors of the source publications. Other study limitations were identified on the basis of a checklist of key RSV-specific potential study limitations. The checklist was developed by a panel of experts in the field of RSV, and epidemiology on the basis of published systematic reviews and reports and the panel’s knowledge and experience. This approach allowed for a more comprehensive assessment of study limitations, rather than relying solely on study authors’ reporting of limitations. Limitations were further characterized as to whether they may contribute to over- or underestimation of RSV epidemiologic estimates.

The rapid review was conducted following good practice guidelines issued by the Cochrane methods group and Joanna Briggs Institute (JBI) [14]. A protocol detailing the study methodology was developed a priori. To ensure completeness and quality of reporting, the principles outlined in the Preferred Reporting Items for Systematic Reviews and Meta-Analyses (PRISMA) guidelines were followed [15]. A PRISMA checklist is presented in the Supplementary Material (File S1). Eligibility criteria are summarized in Table 1. The review was restricted to English-language publications from 2000 onwards, and conference abstracts were not included. While the population of interest in this study is older adults, a broader inclusion criterion of adults aged ≥ 18 years was utilized to increase the sensitivity of the search and ensure all relevant publications were captured.

**Table 1:** Eligibility criteria for inclusion

Element	Inclusion criteria	Exclusion criteria
Population	Adults (≥ 18 years of age)	Paediatric/adolescent patients < 18 years of age
		Mixed-age populations where data for adults were not separately reported
Study design	Primary observational research studies reporting incidence and/or prevalence of RSV in any population	Studies not reporting these outcomes
		Studies with sample size < 50
		Studies investigating a different infection where RSV is investigated only as a co-infection
		Studies in highly specialized populations (e.g. stem cell transplant recipients)
Region	USA, Canada, Europe, Oceania	Studies from other regions
Language	Studies published in English	Studies published in other languages
Time limits	Publication from January 2000 onwards	Published prior to 2000

RSV, respiratory syncytial virus

For publications dated from January 2000 to December 2016, studies meeting the inclusion criteria were identified from a previously published systematic review (Tin Tin Htar et al. [13]). A systematic search strategy was run in Ovid MEDLINE from January 2017 to 3 January 2022 (see Tables S1 and S2 in the Supplementary Material). In addition, the Epistemonikos database (www.epistemonikos.org) was searched for relevant systematic reviews published within the last 3 years and bibliographies were scanned for any further eligible studies. Titles and abstracts were assessed for eligibility by a single reviewer, and relevant full texts were obtained and screened. Any uncertainty on inclusion was resolved by discussion with a second reviewer.

Data on study characteristics, methodology, population, and outcomes of interest were extracted from eligible publications into a bespoke spreadsheet. Data were extracted by one reviewer with key outcomes checked by a second reviewer. Limitations reported by the authors were extracted as free text. Author-reported limitations were grouped by topic for the purposes of evaluation and discussion. Formal quality appraisal with specific quality assessment tools covering all aspects of study methodology was not undertaken. The main reason for this was that no quantitative synthesis was planned, and the focus of the study was on RSV-specific author-reported and other potential limitations which may be subjective and not directly related to the quality of the study.

This article is based on previously conducted studies and does not contain any new studies with human participants or animals performed by any of the authors.

## Results

### Study Inclusion and Characteristics

**Fig. 1:**
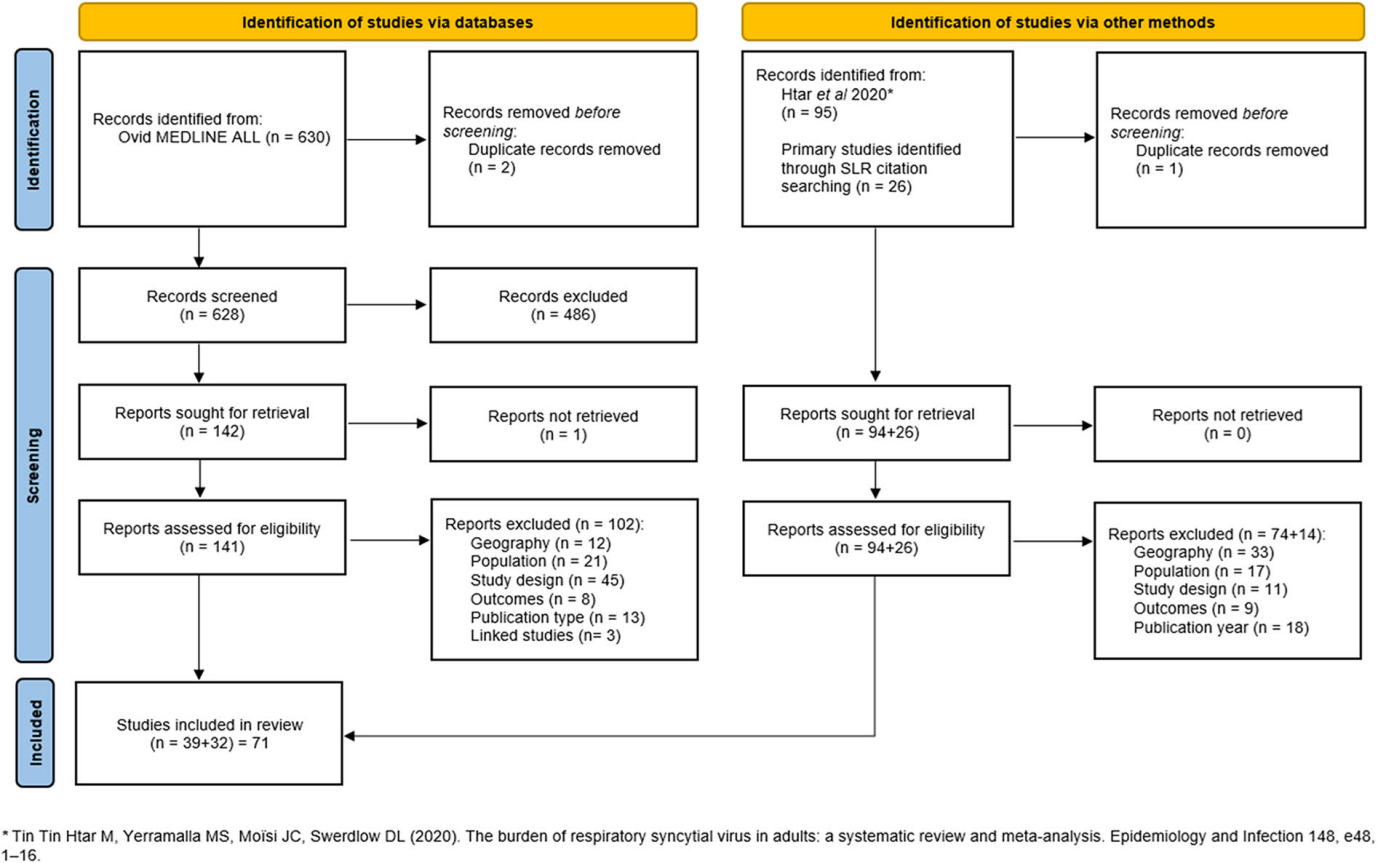
PRISMA flow diagram illustrating the study selection process

A total of 71 publications met the inclusion criteria (see Fig. 1 and Table S3 in the Supplementary Material). Over a third (*n* = 27) were from the USA; the remainder were from European countries, Canada, Australia, and New Zealand. There was wide variation in study methodologies and populations.

Studies were identified as reporting either incidence (*n* = 12), prevalence (*n* = 24) or proportion (*n* = 34) according to the designation given by the study authors; one study reported both incidence and proportion. There was no clear distinction between outcomes described as ‘prevalence’ and those described as ‘proportion’; both described the prevalence of RSV in the study population. Of the studies reporting incidence, all but one [16] dealt with the incidence of symptomatic RSV. Most studies (*n* = 61) were in populations presenting to healthcare providers for respiratory infections or influenza-like illnesses (ILI): 10 in primary, 33 in secondary and 4 in tertiary care; 5 in care homes; and 10 in mixed settings. Additionally, four studies were conducted by laboratories using samples received from a range of sources. Key population characteristics are summarized in Table S4 in the Supplementary Material and are further discussed in the context of the study limitations below.

### Overview of Study Limitations

Author-reported limitations are summarized by topic in Table 2. Other limitations are reported in Table 3.

**Table 2:** Author-reported limitations, by topic

Reported limitation^a^	Examples	No. of reporting limitations (*n* = 71)	Potential bias (under- or overestimation)
Results not generalizable outside study setting/population	Restricted geographic coverage; population restrictions (e.g. hospitalized or medically attended patients only); assessment of single season	29	Under or over
Sampling period	Sampling not tailored to RSV season (e.g. use of influenza season)	18	Under
	Sampling gaps within study period		
Sample size	Small sample size leading to unstable estimates	11	Under or over
Testing method	Quality or sensitivity issues relating to PCR testing	10	Under
	Lack of serology		
Not testing all eligible population	Significant proportion not sampled	10	Under (incidence)
Suboptimal case definitions	Definitions of ARI, ILI or choice of required symptoms for inclusion may miss RSV cases, as RSV may not feature fever or cough	9	Under
Duration between enrolment and viral testing	Viral shedding reduces over time and virus may disappear from sample site	5	Under
Sampling method	Single sampling site (e.g. nasopharyngeal only; serology only)	5	Under
	Suboptimal duration or method of sample storage		

ARI, acute respiratory illness; ILI, influenza-like illness; RSV, respiratory syncytial virus

^a^Only issues specifically reported by study authors as limitations are included

**Table 3:** Occurrence of selected potential limitations (regardless of author reporting)

Potential limitation	*N* with limitation (*n* = 71)	Potential for bias (under- or overestimation)
Single-site sampling (only one site sampled per patient)^a^	62	Under
Upper respiratory tract (nasal/nasopharyngeal/oropharyngeal) only	44	If PCR: virus may no longer be present at the site sampled, for example, have migrated from upper to lower respiratory tract
Lower respiratory tract (sputum, bronchoalveolar lavage) only	1	If serology: acute specimen may be taken too late to capture accurate serologic baseline if patients present for care late
Serum (serology) only	1	If serology: convalescent specimen may be taken too late to capture fourfold rise
Suboptimal case definition	24	Under: not all patients with RSV meet influenza-based or cough-based definitions
Use of ‘influenza-like illness’	18	
Use of ‘acute respiratory infection with cough’	6	
Sampling only in influenza season	13	Under: influenza and RSV seasons both occur during the winter respiratory season but usually only partially overlap
Only a single season assessed	21	Under or over: single season may not be representative due to variability between seasons
Results not stratified by age group within adults	44	In older adults, under: a single rate based on symptomatic infection in all adults will underestimate its incidence in older adults

^a^A total of 16 studies used a variety of sampling sites (one site per patient); 9 used dual sampling (two separate sites per patient, either upper and lower respiratory tract, or respiratory tract and serum)

PCR, polymerase chain reaction; RSV, respiratory syncytial virus

### Limitations Relating to Study Population

#### Symptomatic Versus All Infections

The epidemiological evidence on RSV infection is largely limited to symptomatic cases. Only three studies assessed overall RSV incidence rather than incidence of symptomatic RSV [10, 16]. Hall et al. found that 16% of RSV infections among healthy working adults (*n* = 2960, mean age 30 years) over a 10-year period in the USA were asymptomatic [16]. Falsey et al. studied older (≥ 65 years) and high-risk adults (with chronic lung or heart disease) in the community; serology testing at baseline and end of season and found that 10.7% of 102 diagnosed RSV cases were asymptomatic [10]. Korsten et al. identified 59 RSV cases, of which 7 (11.8%) were identified via serology only and were not reported as experiencing acute respiratory tract illness [7]. In addition, a few studies examined the point prevalence of RSV in asymptomatic control groups that formed part of the study design. All found no [17–19] or very low (*n* = 1) [20] presence of RSV virus in these asymptomatic groups. Together, these findings suggest that failure to evaluate asymptomatic groups results in a 10–16% underestimation of infection incidence; however, for disease burden estimates, asymptomatic infections are usually not included.

#### Medically Attended Versus Non-attended Cases

Restriction of the study to medically attended patients was frequently cited by authors as a limitation, as not all patients will seek healthcare. Only nine studies were set in the community, that is, in patients who had not presented to healthcare when tested for RSV, [3, 7, 10, 16, 21–25] and the majority of these required a symptomatic trigger (e.g. patients had to call the study team if they had respiratory symptoms). Three community studies provide evidence on the impact of only including medically attended cases, showing that 52–69% of symptomatic patients with RSV do not seek medical care [7, 10, 21]. Although limited by small case numbers, these findings suggest that studies sampling only medically attended patients will underestimate the number of symptomatic RSV infections.

Most studies (*n* = 62) were in medically attended populations; these are the most relevant group for assessing health–economic burden, although presenteeism and absenteeism due to symptomatic RSV infection without healthcare attendance could have a significant impact on productivity costs (indirect costs). Of these, 36 were exclusively in hospitalized populations. The latter was frequently cited as a cause of underestimation of the full case burden, as non-hospitalized patients would be missed. Knowledge of the percentage of community and primary care patients with RSV who go on to be hospitalized would aid interpretation of these studies. Sundaram et al., reporting on adults aged ≥ 50 in a US influenza vaccine study, reported that 17 of 204 (8.3%) RSV cases identified in the outpatient setting were hospitalized. Also in the USA, Belongia et al. found that 11.9% (29 of 243) of adults aged ≥ 60 years who sought outpatient care for RSV-related acute respiratory illness (ARI) were hospitalized [26]. These findings suggest that deriving RSV incidence estimates from hospitalized populations may underestimate the number of medically attended cases, even among older adults.

### Limitations Related to Case Definitions

Almost all studies required the presence of defined symptoms (the ‘case definition’) as a condition of inclusion in the population to be tested for RSV. A number of different case definitions were used: ILI (*n* = 14), ARI (*n* = 22), severe acute respiratory illness (SARI) (*n* = 7), respiratory symptoms (*n* = 10), respiratory tract infection (*n* = 8), and ARI with cough (*n* = 6). Definitions within these categories were not consistent, and limitations relating to case definitions were cited by a number of authors.

#### Use of ILI and Fever-Based Definitions

The use of influenza-based definitions and/or a requirement for fever carry considerable potential for underestimation because a significant proportion of RSV-infected older adults do not display fever [27, 28]. Elderly adults have a reduced likelihood of fever in acute infections because of diminished febrile response and/or lower baseline body temperature [29]. The community surveillance study by Hall et al. found that only 28% of 177 symptomatic RSV cases displayed fever [16]. In a study of community-based adults aged ≥ 65 years taken from a vaccine trial, Falsey et al. used an ILI definition that did not require fever, and found that fever was only present in 56.4% (95% CI 39.6–72.2%) of the 39 RSV cases identified [30]. Furthermore, using symptom data from a European prospective cohort study of community-dwelling adults aged ≥ 60 years, Korsten et al. [28] found that the World Health Organization (WHO) ILI definition had a sensitivity of only 11% for identifying patients with PCR-confirmed RSV-acute respiratory tract infection (ARTI), leading to a ninefold underestimation of RSV. A modified ILI definition, requiring feeling feverish rather than measured fever, also had low sensitivity (33%). The low sensitivities were primarily related to the inclusion of fever because in the remainder of confirmed RSV–ARTI patients, fever/feeling of fever was not observed.

Use of fever-based definitions is common because many RSV studies are embedded in influenza programs or studies. Among the larger studies we identified, Descamps et al. (France), [31] Pellegrinelli et al. (Italy), [5] Saez-Lopez (Portugal, two studies) [32, 33], Subissi et al. (Belgium) [34, 35], Tramuto et al. (Italy) [36], Van Beek et al. (theNetherlands) [22], Varghese et al. (Australia) [37], Belongia et al. (USA) [26] and Zambon et al. (UK) [38] all used ILI or SARI definitions that included fever. In many cases the authors report this as a limitation that may have led to RSV being underestimated. Loubet et al. used another variation of ILI definition which required at least one systemic symptom (fever ≥ 38 °C, headache, myalgia or malaise), and at least one of cough, sore throat or dyspnoea. They acknowledged that even this modified definition captures only a subset of RSV infections [39]. Saez-Lopez et al. [33] assessed the performance of three case definitions and concluded that the EU ILI case definition currently in use for RSV surveillance in Portugal is not suitable.

#### Use of ARI Definitions

The most common symptom requirement was ARI (*n* = 22) or similar [respiratory symptoms (*n* = 10) or respiratory tract infection (*n* = 8)]. Definitions of ARI varied and were often narrower than the ARI definition suggested by the WHO for community-based RSV surveillance, which defines respiratory infection simply as ‘at least one of shortness of breath or cough; sore throat; and coryza’ (runny nose/sneezing) [27]. Branche et al. [40], in a prospective study of hospitalized patients, extended their screening case definition beyond ARI alone to include patients hospitalized for heart failure, chronic obstructive pulmonary disease (COPD) or asthma who had ARI in the previous 14 days. The objective was to ensure inclusion of RSV-associated exacerbations, and these broad criteria were cited as strengths by the authors.

### Limitations Related to Seasonality

#### Sampling Period Not Tailored to RSV Season

RSV has a distinctly seasonal circulation pattern [1, 41] which in any given year may only partially overlap with the influenza season. This was illustrated by data from French surveillance networks reported by Loubet et al. [39], which showed clear separation of the RSV and influenza peaks. A large proportion of RSV cases were seen whilst influenza circulation was still low. Subissi et al. tested the capability of the Belgian SARI network, which is based around influenza surveillance, to contribute to RSV surveillance. They began surveillance earlier (week 40) to reflect the RSV season and used a modified SARI definition. They found that peak RSV incidence occurred at week 49, and cases had already fallen considerably by the start of influenza surveillance at week 2 of the following year [34]. Patterns vary around the world [41], but in general sampling that is restricted to the influenza season will miss a significant number of RSV cases and will underestimate the case burden of RSV.

Approximately half of the identified studies explicitly reported that their sampling was seasonal in some way. Seasons reported included the influenza season (*n* = 13), the respiratory virus season (*n* = 1) and the winter season (*n* = 15); only 9 studies [7, 16, 32, 34, 40, 42–45] specifically matched their sampling to the RSV season, or appeared to do so from the period reported (roughly October to April in the Northern Hemisphere). Awareness of the limitations around non-RSV-specific sampling periods was high. A number of the larger, more recent studies we identified were carried out in the influenza season only, and the authors acknowledged this as a limitation [35, 36, 39, 46, 47]. Many of these studies also used influenza-based ARI and SARI definitions; a limitation acknowledged again by the authors in most cases.

#### Sampling a Single Season

The level of RSV activity within the RSV season can vary from year to year [41]. Some countries (e.g. Scandinavian countries, Germany) display a biennial pattern of major followed by minor outbreaks, while others (e.g. Spain, the UK) have a more stable annual epidemic [41]. Branche et al. found that RSV incidence was ‘remarkably consistent’ over the three seasons in the USA [40]. Also in the USA, McClure noted that incidence of medically attended RSV was similar across four seasons [48]. In contrast, Korsten et al. reported substantial variation in RSV incidence between the two seasons studied [7].

Because of the potential for variation, data on epidemiology from a single season should be treated with caution. Widmer et al. [49], Falsey [30] and Self [17] were among those studies that examined only one season and cited this as a limitation. Caution should also be applied to studies that encompass the period of the coronavirus disease 2019 (COVID-19) pandemic, as they are unlikely to be typical of normal years. Stamm et al. [50] compared RSV incidence in emergency room patients before and during the pandemic and found that the RSV positivity rate fell to zero during 2020–2021. They suggested this might be due to public health measures and infection control behaviours.

### Limitations around subgroups reported

Over half of studies (*n* = 44) reported on an all-age adult sample and not specifically on older adults. Failure to distinguish older adults is a limitation if the study is to be used to assess burden and/or inform vaccine strategy, as both the incidence of symptomatic infection and the risk of significant morbidity from RSV increase markedly with age [1, 45, 51]. Data from an all-age adult sample will therefore underestimate incidence in the most at-risk population.

People with chronic health conditions, particularly lung and cardiovascular conditions, are at particularly high risk from RSV [1]. Only about a third of studies reported the prevalence of comorbidities in the included population. Failing to explicitly consider patients with comorbidities may be considered a limitation when seeking to ascertain which groups have the greatest incidence and burden of RSV. We identified ten studies specifically designed to assess patients with comorbidities [3, 10, 24, 52–58], although most were not specifically designed around RSV. Branche et al. reported that RSV incidence rates in hospitalized adults with broadly defined ARI were up to 13-fold higher in patients with exacerbation of underlying cardiopulmonary conditions. As the authors noted, this illustrates the importance of assessing the burden of RSV in groups with underlying conditions to inform vaccine policy; currently, testing for RSV in exacerbations is not routine practice [40]. Not including immunocompromised patients in sampling could also contribute to underestimation of RSV: Reckziegel et al. found that immunocompromised adults had a higher RSV prevalence than non-immunocompromised (6.4% versus 2.4%, *p* = 0.078) [58].

### Limitations around Sampling and Testing Methodology

The great majority of studies were based on sampling of the upper respiratory tract only (nasal, nasopharyngeal or oropharyngeal swabs). Few included sampling of sputum or other lower respiratory tract specimens, and many of those that did were based on samples examined by public health laboratories and lacked contextual data. Upper respiratory tract sampling can miss infections: adults with RSV shed the virus for only 3–4 days, [1] and in many studies this period may be over before the patient is tested. In more severe disease, RSV migrates from the upper to the lower respiratory tract during the illness, again meaning that lower respiratory tract infections may be missed [59].

Paired serology (sampling of RSV antibody titres in the blood at baseline and after symptoms) is a way of detecting infection outside of the virus shedding period, [60] but only ten of the identified studies [3, 4, 7, 54, 61–66] used serology. Zhang et al. found adding paired serology to reverse transcription polymerase chain reaction (RT-PCR) of naso-/oropharyngeal swabs increased RSV case detection by 28.6% (95% CI 5–82) in adults aged 18–64 and 50.0% (95% CI 9–167) in those aged ≥ 65 years [60]. Serology also has potential for underestimation as some patients do not seroconvert after infection (seroconversion defined as a ≥ fourfold increase antibody). Korsten et al. used both PCR and paired serology (pre- and post-season) in their study of community-dwelling older adults and noted that antibody decay could have occurred before the post-season serology sample was taken, which would lead to underestimation [7]. McLaughlin et al. reviewed US-based studies that paired nasal or nasopharyngeal sampling with either serology (fourfold rise defined as positive) or sputum sampling, and found that adding either of these techniques identified a median of 1.5 times more RSV infections in medically attended adults than nasal/nasopharyngeal sampling alone [59]. A systemic literature review and meta-analysis by Onwuchekwa et al. found RSV detection increased by 52% by adding RT-PCR of sputum, 28% by adding RT-PCR of oropharyngeal swab, and 42% by adding serology testing of paired specimens, compared with nasopharyngeal/nasal swab alone [67]. In addition, Ramirez et al. examined the synergistic effect of adding multiple specimen types to nasopharyngeal swab and found a nearly fourfold increase in RSV detection associated with adding sputum, saliva and paired serology [68].

### Sample size and low case numbers

Small sample size was reported as a study limitation by some authors due to the associated uncertainty. Few studies reported confidence intervals or other measures of uncertainty, which constitutes a limitation of much of the evidence base. Extrapolation to annual incidence from a small number of RSV cases could introduce bias. Branche et al. cited the fact that they had extrapolated from 1099 RSV-positive patients as a strength of their study [40], noting that some studies (e.g. Widmer [47, 49]) have used as few as around 30 RSV cases. However, the incidence rates calculated by Branche et al. were similar to those from Widmer.

### Limitations in Generalizability

Generalizability outside of the specific study setting (e.g. geographical area, study population) was the most frequently cited limitation by authors. The study by Falsey et al. was unusual in that it covered 15 countries, with subjects taken from an influenza vaccine trial, in persons aged ≥ 65 years with moderate-to-severe ILI. Prevalence of RSV ranged from 0–17.1% (12/70) by country. The differences may be partially attributable to low ILI case numbers registered in some countries, but seasonal variation between countries and the effects of different household structures were also cited as potential contributors to national differences. The authors noted that studies in individual countries over multiple seasons would be required to better understand regional variation. Incidence may also vary regionally within countries: for example, rural areas may have lower transmission and therefore lower incidence of RSV than densely populated areas, particularly areas with crowded housing or multi-generational households [21, 26, 40]. Branche et al. suggested that local social and population determinants of RSV risk could be considered in design of clinical trials [40]. Widmer cited their restriction to one geographical location as a limitation of their study [47].

Generalizability from one healthcare setting to another is limited. Wansaula et al. noted that their study setting in five large referral hospitals biased their sample to more severe viral illnesses presenting later in the course of disease [69]; this has the potential to underestimate the overall symptomatic incidence of a given infection. The possibility of referral centre bias was also cited by Smithgall et al. [21] and Loubet [39]. Subissi cited sampling bias relating to surveillance by general practitioners, stating that in Belgium general practitioners (GPs) are less likely to have older people as patients compared with the general population, and less likely to sample them [35]. Jackson et al. conducted their study in a privately insured cohort in the USA and noted that such individuals may have different illness thresholds for seeking medical care than other populations [51]. These examples illustrate that health system features can cause sampling bias that may cause variability in measured estimates, and such biases will differ between countries.

Some studies reporting prevalence of RSV are from virological surveillance laboratories. The utility of these data are limited by the absence of a clear denominator and lack of data on the number of patients tested, the criteria used for testing, or characteristics of included patients [70]. Subissi et al. is an example of a study that retrospectively calculated incidence from sentinel surveillance data and reported estimates much lower than those from prospective studies [35].

### Use of Retrospective Database Studies and Disease Codes

In clinical practice, the primary reason for underestimation of symptomatic RSV infection is lack of routine testing for RSV in patients with ARI [2]. This limitation affects studies based on health claims databases, as diagnostic codes for RSV are only present if testing was carried out. A small number of studies based on retrospective health claim databases were identified but were excluded from the review as they focused on hospitalization and other outcomes and did not report original data on RSV incidence [6, 71–73]. These studies estimated incidence of RSV-related healthcare utilization/hospitalization on the basis of the presence of RSV-specific International Classification of Disease (ICD) codes, in some cases supplemented by applying data from proportion studies or surveillance programs to attribute a percentage of other related codes to RSV. These studies are likely to significantly underestimate both incidence and healthcare utilization rates because of lack of testing for RSV [71]. One study of US hospitals found that in more than 75% of hospitals, less than 25% of hospitalizations for lower respiratory tract infections involved RSV testing [74].

## Discussion

In this review, we summarize limitations of published studies estimating the incidence and prevalence of RSV. Because of the wide heterogeneity in study methodologies, populations, and seasons, comparison of incidence estimates between studies should be treated with caution. It is therefore difficult to gauge the magnitude of under- or overestimation associated with a particular limitation, making the overall effect size unclear. However, it is reasonable to conclude that a significant proportion of studies underestimate RSV case numbers because of the widespread use of case definitions and/or sampling periods on the basis of influenza detection or otherwise not specifically tailored to RSV, the reliance on RT-PCR testing of upper respiratory tract samples as the only means of detection, and the lack of routine testing for RSV in clinical practice.

Variations in case definitions (the symptoms required for inclusion in the population that is tested for RSV) are a source of heterogeneity in RSV incidence estimates and can result in underestimation because RSV cases are missed. In its RSV surveillance pilot, launched in 2016 [75], the WHO used an extended definition of SARI for hospital-based surveillance and a modified ARI definition for community-based surveillance. These definitions do not require fever to identify a suspect case. The WHO notes that their use substantially increased the number of RSV infections detected compared with its ILI definition or ILI-based SARI definition, and that this is important when surveillance data are used to estimate disease burden [27, 75]. Other authors have also confirmed the low sensitivity for RSV of influenza-based and fever-based case definitions [28, 33].

The majority of studies did not report tailoring sampling to the RSV season. This reflects two features of the evidence base. Firstly, many studies are carried out by influenza surveillance networks during their normal operation period, which is often not aligned with the RSV season. Many combine this with influenza-based case definitions; together this is likely to result in large numbers of cases being missed. Secondly, many do not have the study of RSV as their main objective; rather, RSV is one of several viruses screened for. Thus the study design may not be optimized for RSV detection.

The great majority of RSV case estimates are based solely on PCR testing of upper respiratory tract swabs, which produces lower detection rates than when combined with sputum sampling [59]. However, not all patients can produce sputum samples, and other lower respiratory tract sampling (e.g. bronchoalveolar lavage) is invasive and is often not a practical study option, as obtaining consent for research purposes is difficult. Few studies carried out serology, which detects RSV infections missed by PCR [59, 60]. Although it adds to the study burden, paired serology can play a complementary role to PCR in assessing the incidence of RSV [67]. This is still possible in vaccinated populations as assays can distinguish between natural and vaccine-induced antibodies [68]. However, even combined serology and PCR can still under-report as the timing of acute and convalescent samples must be optimal. A multiplier reflecting the percentage of additional cases likely to be picked up if serology or sputum sampling had been performed could be a useful approach, as used in a recent meta-analysis of US RSV disease incidence rates by McLaughlin et al. [59].

Placebo arms of phase III RSV vaccine trials offer a potentially valuable source of burden data as they incorporate comprehensive prospective disease surveillance, but trial participants differ from the general population in important ways that would bias the rate of illness downwards. These include exclusion of immunocompromised persons, lower frequency of comorbid conditions, and the healthy volunteer effect, in which persons volunteering for trials are generally healthier and have lower mortality than the general population.

Our study was dependent to some degree on author reporting of limitations, although the presence of specific RSV-related limitations was assessed as far as possible within the constraints of the information available. A limitation of our review was its restriction to English-language publications from Europe, North America and Oceania; RSV estimation in other regions may be subject to different limitations. We did not aim to synthesize incidence or prevalence estimates.

### Evaluating and Designing Studies

Authors should report clearly on the key items that allow studies to be evaluated, and should discuss limitations and their likely effect on estimates. Reporting in the studies we identified was inconsistent on items such as proportion of eligible patients sampled or rationale for choice of sampling period. Ideally, appropriate quantitative adjustments should be made to compensate for limitations that affect the accuracy of estimates. In addition, it is important that studies should report specifically on older adults and/or those with cardiopulmonary comorbidities, as these groups account for by far the largest proportion of the health and economic burden of adult RSV infection.

To ensure representativeness and generalizability to the intended target population, studies should ideally start with defining a formal sampling frame and randomly sample a sufficient number of sites from the pool of all eligible locations, potentially stratified by risk factors of RSV incidence, such as rural–urban classification. Depending on the desired level of precision, a random subset of patients attending at the selected sites could be invited for further participation. When aiming to capture incidence of RSV cases that do not result in healthcare attendance as well, for example, to quantify potential productivity losses associated with RSV infections including those not resulting in healthcare attendance, the sampling framework could be built around households as done for severe acute respiratory syndrome coronavirus 2 (SARS-CoV-2) surveillance [76]. Potential non-response of sites or eligible participants could be accounted for using survey weights or multilevel regression and post-stratification [76].

Key points for consideration when evaluating and designing studies are proposed below (Table 4).

**Table 4:** Points to consider when evaluating and designing RSV epidemiology studies in adults

Population and case definition	
What is the appropriate population for the intended use of the data (all symptomatic patients, primary care/outpatient cases, hospitalized cases)?	
Does reporting of incidence/prevalence distinguish between the overall adult population and higher risk groups (older adults, those with comorbidities)?	
Is the case definition for screening appropriate for RSV in adults?	
Sampling	
Does the sampling period capture the full local RSV season?	
If only one season is reported, how much variation between seasons is typical in the country in question?	
Does the sampling period include the COVID-19 pandemic?	
What proportion of eligible sites and/or patients were sampled? Is there potential for under-reporting or sampling bias?	
What was the delay between symptom onset and sampling? Could patients have stopped shedding virus by the time of sampling?	
Is the sample size adequate? Is the number of RSV cases detected sufficient to support any extrapolations made?	
Reporting and adjustment for limitations	
If the study relies on single-site sampling (e.g. PCR testing of upper respiratory tract samples), consider applying a multiplier on the basis of published estimates [59, 60] to reflect likely proportion of cases missed	
If sampling is not year round, are adjustments made to account for RSV activity outside the sampling period?	
Are appropriate measures of uncertainty reported?	
Is reporting stratified by age range (specifically older adults age 65+) and/or comorbidity status to allow for assessment of burden in the groups most vulnerable to severe illness from RSV?	
In retrospective claim database studies, have adjustments been made to compensate for low rates of RSV testing in clinical practice?	
Are study limitations reported and their potential impact on accuracy and generalizability discussed? Have appropriate quantitative adjustments been made to compensate for likely under-reporting and selection bias?	

It must be noted that the present study is not exempt of limitations. A rapid literature review methodology was followed rather than conducting a full systematic literature review. Although in a rapid review a systematic approach is used to conduct the search, screen, and extraction, some steps within the evidence synthesis process may be simplified or omitted. No comprehensive grey literature search was conducted, and systematic searches were limited to most relevant databases only (MEDLINE for primary studies and Epistimonikos for relevant systematic reviews). To minimize the impact of these limitations and ensure that key literature was captured, an information specialist was consulted to design the search strategy, and bibliographies of relevant systematic reviews were scanned for additional studies that may not have been captured through database searches. Finally, as previously stated, several adult RSV vaccines are currently under development, and clinical studies are ongoing to assess their efficacy and safety. Though this suggests that new vaccine options will likely be available in the near future, results from these studies are largely unreported, and consequently, it was not possible to provide a large evidence base to support RSV vaccine efficacy in the present review.

## Conclusion

A significant proportion of studies are likely to underestimate the incidence of RSV infection in older adults, although the effect size is unclear and there is also potential for overestimation. Underestimating the case burden of RSV in older adults will in turn underestimate the potential health benefits that can be gained from a vaccination program. The effectiveness and public health impact of vaccines will also be underestimated, as the recorded reduction in cases will be based on the incomplete proportion of cases captured prior to the vaccination program. Well-designed studies, together with increased testing for RSV in patients with ARI, are required to accurately capture both the burden of RSV and the potential public health impact of vaccines.

## Supplementary Information

Below is the link to the electronic supplementary material.

**Table S1**: Ovid MEDLINE search strategies. **Table S2**: Supplementary Table S4 Epistemonikos search strategy. **Table S3**: Study characteristics of included studies. Abbreviations: ARI, acute respiratory illness; RSV, respiratory syncytial virus; ILI, influenza-like illness; SARI, severe acute respiratory illness; RTI, respiratory tract infection; COPD, chronic obstructive pulmonary disease; AECOPD, acute exacerbation of chronic obstructive pulmonary disease; LRTI, lower respiratory tract infection; CAP, community-acquired pneumonia; CCU, critical care unit; MV, mechanical ventilation; ARTI, acute respiratory tract infection. Table S4: Population characteristics of included studies. Abbreviations: AECOPD, acute exacerbation of chronic obstructive pulmonary disease; CAP, community acquired pneumonia; COPD, chronic obstructive pulmonary disease; IQR, interquartile range; NR, not reported; SD, standard deviation * Children or adolescents were also evaluated in some studies. ^†^During the 2004–2005 season, patients aged 50–64 were eligible if they had at least 1 high-risk chronic medical condition. **File S1:** PRISMA 2009 Checklist. (PDF 479 KB)

## Data Availability

All data generated or analyzed during this study are included in this published article and supplementary information.
