# Supplementary material for: Incidence of Respiratory Syncytial Virus Infection in Older Adults: Limitations of Current Data
Source: Infect Dis Ther. 2023 May 23;12(6):1487–504. doi: 10.1007/s40121-023-00802-4 (PMC10262134; doi:10.1007/s40121-023-00802-4)
Supplement: Supplementary file 1 — Table S1: Ovid MEDLINE search strategies. Table S2: Supplementary Table S4 Epistemonikos search strategy. Table S3: Study characteristics of included studies. Abbreviations: ARI, acute respiratory illness; RSV, respiratory syncytial virus; ILI, influenza-like illness; SARI, severe acute respiratory illness; RTI, respiratory tract infection; COPD, chronic obstructive pulmonary disease; AECOPD, acute exacerbation of chronic obstructive pulmonary disease; LRTI, lower respiratory tract infection; CAP, community-acquired pneumonia; CCU, critical care unit; MV, mechanical ventilation; ARTI, acute respiratory tract infection. Table S4: Population characteristics of included studies. Abbreviations: AECOPD, acute exacerbation of chronic obstructive pulmonary disease; CAP, community acquired pneumonia; COPD, chronic obstructive pulmonary disease; IQR, interquartile range; NR, not reported; SD, standard deviation * Children or adolescents were also evaluated in some studies. †During the 2004–2005 season, patients aged 50–64 were eligible if they had at least 1 high-risk chronic medical condition. File S1: PRISMA 2009 Checklist. (PDF 479 KB) [file 40121_2023_802_MOESM1_ESM.pdf]

Supplementary Material

Incidence of respiratory syncytial virus infection in older adults: limitations of current data

Authors: Mark H Rozenbaum,[1] Elizabeth Begier,[2*] Samantha Kurosky,[3] Jo Whelan,[4] Danai Bem,[4] Koen B Pouwels,[5] Maarten Postma,[6] Louis Bont[7]

[1] Pfizer Inc, Capelle a/d IJssel, The Netherlands

[2] Pfizer Vaccines, Dublin, Ireland

[3] Pfizer Inc, New York, NY, USA

[4] HEOR Ltd, Cardiff, UK

[5] University of Oxford, Oxford, UK

[6] University of Groningen, Groningen, the Netherlands

[7] University Medical Center Utrecht, Utrecht, the Netherlands

*Corresponding author

Email: Elizabeth.Begier@pfizer.com

Address: Pfizer Vaccines, Dublin, Ireland

S1 Table. Ovid MEDLINE search strategies.

Ovid MEDLINE(R) ALL: 1946 to January 03, 2022
Searched: 04.01.22

#	Search Terms	Results
**Disease terms**		
**1**	exp Respiratory Syncytial Viruses/ or (respiratory syncytial virus$ or human orthopneumovirus$ or respirosyncytial virus$ or respiro-syncytial virus$ or RS virus$ or RSV).ti,ab.	20,463
**Epidemiology terms**		
2	Incidence/ or Epidemiology/ or Prevalence/	595,572
3	(Incidence or epidemiolog$ or prevalence or ?etiology or episode$ or distribution$).ti,ab.	2,981,801
**4**	or/2-3	3,139,974
**Study design terms***		
5	exp Cohort studies/ or exp Longitudinal Studies/ or exp Prospective Studies/ or exp Follow-Up Studies/ or exp Case-Control Studies/ or exp Case Reports/ or Observational study/	4,681,530
6	(cohort$ or (case$ adj2 control$) or (case$ adj2 series) or (case$ adj2 report$) or (case$ adj2 stud$) or (observation$ adj2 (stud$ or report$)) or surveillance).tw.	1,988,947
**7**	or/5-6	5,494,502
**Combine terms** (Disease AND epidemiology AND study design)		
**8**	1 and 4 and 7	1,946
**Limit to English language and publication date**		
**9**	limit 8 to (english language and yr="2017 -Current")	630
**Total no. of studies identified**		**630**
*Observational filter adapted from: Fraser C, Murray A, Burr J. Identifying observational studies of surgical interventions in MEDLINE and EMBASE. BMC Medical Research Methodology. 2006;6(1):41.		

S2 Table. Supplementary Table 4 Epistemonikos search strategy.

Epistemonikos (www.epistemonikos.org): searched for relevant systematic literature reviews, up to 4 January 2022
Searched: 7.2.22

Search	Results
(title:((title:("respiratory syncytial virus*" OR "human orthopneumovirus*" OR "respirosyncytial virus*" OR "respiro-syncytial virus*" OR "RS virus*" OR RSV) OR abstract:("respiratory syncytial virus*" OR "human orthopneumovirus*" OR "respirosyncytial virus*" OR "respiro-syncytial virus*" OR "RS virus*" OR RSV)) AND (title:(Incidence OR epidemiolog* OR prevalence OR aetiology OR etiology OR episode* OR distribution*) OR abstract:(Incidence OR epidemiolog* OR prevalence OR aetiology OR etiology OR episode* OR distribution*)) AND (title:((cohort* OR ((case*) AND (control* OR series OR report* OR stud*)) OR ((observation*) AND (stud* OR report*)) OR surveillance)) OR abstract:((cohort* OR ((case*) AND (control* OR series OR report* OR stud*)) OR ((observation*) AND (stud* OR report*)) OR surveillance)))) OR abstract:((title:("respiratory syncytial virus*" OR "human orthopneumovirus*" OR "respirosyncytial virus*" OR "respiro-syncytial virus*" OR "RS virus*" OR RSV) OR abstract:("respiratory syncytial virus*" OR "human orthopneumovirus*" OR "respirosyncytial virus*" OR "respiro-syncytial virus*" OR "RS virus*" OR RSV)) AND (title:(Incidence OR epidemiolog* OR prevalence OR aetiology OR etiology OR episode* OR distribution*) OR abstract:(Incidence OR epidemiolog* OR prevalence OR aetiology OR etiology OR episode* OR distribution*)) AND (title:((cohort* OR ((case*) AND (control* OR series OR report* OR stud*)) OR ((observation*) AND (stud* OR report*)) OR surveillance)) OR abstract:((cohort* OR ((case*) AND (control* OR series OR report* OR stud*)) OR ((observation*) AND (stud* OR report*)) OR surveillance)))))	834
Filtered to systematic reviews, 2019-2022	21

S3 Table. Study characteristics of included studies.

Author (year)	Country	Study design	Setting	Study period	Outcome	Symptoms required for inclusion
Belongia (2018)[1]	USA	Prospective, single centre	Primary and secondary care	Influenza seasons, 2004-2016	Incidence	Fever/feverishness or cough and ARI with cough (2011-2016)
Bozio (2018)[2]	USA	Prospective, multicentre	Secondary care	January 2010-June 2012	Proportion	ARI and radiographically confirmed pneumonia
Branche (2014)[3]	USA	Prospective, single centre	Secondary care	4 winter seasons (November-May), 2008-2012	Proportion	ARI
Branche (2021)[4]	USA	Prospective, multicentre	Secondary care	3 RSV seasons (October-April), 2017-2020	Incidence	≥2 ARI symptoms: fever ≥37.8°C or feeling feverish, new or worsening cough, new or worsening sputum production, new or worsening dyspnea, sore throat, runny nose/nasal congestion, and body aches or exacerbation of underlying cardiopulmonary disease preceded by ARI symptoms in the past 14 days
Brittain-Long (2010)[5]	Sweden	Prospective, multicentre	Secondary care	2 winter seasons (October-April), 2006-2008	Proportion	Acute RTI: at least two of coryza, congestion, sneezing, sore throat, odynophagia, cough, chest pain, shortness of breath or fever, with no other explanation
Camargo (2008)[6]	USA	Prospective, multicentre	Secondary care	Winter season, December 2003-April 2004	Prevalence	AECOPD and any combination of increased cough, purulent sputum, dyspnea, fever, and chest congestion present for 10 days and a cigarette smoking history of 20 pack-years or more
Carrat (2006)[7]	France	Prospective, single centre	Secondary care	2 winter seasons, (October 2002-March 2003, November 2003-February 2004)	Prevalence	Chronic cardiac or pulmonary disorders admitted to a CCU for acute respiratory or cardiac failure
Charles (2008)[8]	Australia	Prospective, multicentre	Secondary care	4 sites: 3 southern hemisphere winters, June 2004-September 2006<br>2 sites: June 2004-June 2005	Proportion	CAP: chest radiograph within 24 hours after hospital admission demonstrating features consistent with acute pneumonia and at least 2 symptoms consistent with pneumonia of fever or hypothermia, rigors, sweats, new cough with or without sputum, chest discomfort, or new-onset of dyspnea
Chasqueira (2018)[9]	Portugal	Prospective, multicentre	Elderly care centres	November 2013-April 2014	Prevalence	ARI: sudden onset of symptoms, at least one respiratory symptom of cough, sore throat, shortness of breath and coryza, and a clinician’s judgement that the illness was due to an infection

Ciotti (2020)[10]	Italy	Retrospective, single centre	Secondary care	October 2016-March 2019	Proportion	Hospitalised with respiratory symptoms
Civljak (2019)[11]	Croatia	Retrospective, single centre	Secondary care	January 2016-June 2018	Prevalence	ARI symptoms for at least 7 days
Creer (2005)[12]	UK	Prospective, multicentre	Primary care	May 2000-April 2001	Proportion	Acute LRTI: new or worsening cough and at least one other lower respiratory tract symptom for which there was no other explanation, present for ≤ 21 days
De Francesco (2021)[13]	Italy	Retrospective, single centre	Secondary care	January 2017-May 2021	Prevalence	Respiratory symptoms: at least one of shortness of breath, sore throat, cough and fever ≥37.5°C
De Serres (2009)[14]	Canada	Prospective, multicentre	Secondary care	2 winter seasons, January 2003- May 2003, January 2004-May 2004	Proportion	AECOPD: increase of respiratory symptoms (dyspnea, cough, and sputum) requiring an unscheduled medical visit <10 days after onset of symptoms
Descamps (2021)[15]	France	Prospective, multicentre	Secondary care	2 winter seasons, 2017-2019	Prevalence	ILI: at least one systemic symptom of fever or feverishness, malaise, headache, myalgia or deterioration of general condition (asthenia or loss of weight or anorexia or confusion or dizziness) and at least one respiratory symptoms cough, sore throat or shortness of breath with onset <7 days before swabbing and hospitalised for at least 24 hours
Diaz-Decaro (2018)[16]	USA	Prospective, multicentre	Skilled nursing home facilities (SNFs)	May 2015-July 2015	Prevalence	ARI
Dimopoulos (2012)[17]	Greece	Prospective, single centre	Tertiary care	January 2008-December 2009	Proportion	AECOPD: a change in "baseline" dyspnea, cough and sputum (beyond the normal day-to-day variation) usually requiring a modification in regular medication of a stable COPD course
Falsey (2005)[18]	USA	Prospective, multicentre	Community (healthy elderly persons and high-risk adults)	4 winter seasons, 1999-2003	Proportion	Symptoms of respiratory illness or worsening cardiopulmonary symptoms, nasal congestion, sore throat, hoarseness, new or worsening cough, sputum production, and dyspnea with or without fever
Falsey (2006)[19]	USA	Prospective, multicentre	Secondary care	RSV season (January-April), 2004	Proportion	Respiratory illness: one or more of nasal congestion, sore throat, hoarseness, cough, sputum production, dyspnea, wheezing, pleuritic chest pain and myalgias

Falsey (2008)[20]	USA	Prospective, multicentre	Long-term care facilities	1998-2000	Proportion	Respiratory symptoms
Falsey (2014)[21]	Multi-country	Prospective, multicentre	Community	November 2008-April 2009	Prevalence	Moderate-to-severe ILI: associated with pneumonia (based on signs and symptoms with a chest radiograph demonstrating a new or progressive infiltrate), or with hospitalisation, or with a maximum (the highest total score achieved on days 0–14) daily Influenza Symptom Severity (ISS) score >2
Galli (2020)[22]	Italy	Prospective, multicentre	Primary care and healthcare workers	Influenza season, 2018 (week 46)-2019 (week 17)	Proportion	ILI: sudden onset of symptoms, with at least one systemic (fever or feverishness, malaise, headache, and myalgia) and one respiratory (cough, sore throat, and shortness of breath) symptom
Gaymard (2018)[23]	France	Retrospective, single centre	Virology laboratory	4 RSV seasons, September 2010-April 2014	Prevalence	ILI or RTI
Gimferrer (2019)[24]	Spain	Prospective, single centre	Secondary and tertiary care	October 2013 (week 40)-March 2018 (week 9)	Prevalence	clinical suspicion of viral respiratory infection
Glezen (2000)[25]	USA	Prospective, multicentre	Secondary care	July 1991-June 1995	Proportion	Acute respiratory conditions: pneumonia, tracheobronchitis, bronchiolitis, croup, exacerbations of asthma or COPD, and congestive heart failure
Hall (2001)[26]	USA	Prospective, multicentre	Community (healthy working adults)	RSV seasons, 1975-1995	Incidence	Healthy working adults sampled regardless of symptoms
Hause (2019)[27]	USA	Prospective, single centre	Tertiary care (obstetric/ gynaecological clinic)	November 2015-May 2016	Proportion	Pregnant women with an ARI
Hirve (2020)[28]	Multi-country	Multicentre	Primary care	November 2017-June 2018	Proportion	ARI (outpatient): onset within past 10 days and at least one of cough, sore throat, shortness of breath or runny nose<br>SARI (in patient): overnight hospitalisation and onset within past 10 days of cough or shortness of breath<br>ILI: onset of cough within past 10 days
Hutchinson (2007)[29]	Australia	Prospective	Community	July-December 2003 and August 2004-December 2005	Prevalence	COPD exacerbations: type I, an increase in dyspnoea, sputum volume and sputum purulence for more than 24 hours, Type II, any two of the above symptoms and Type III, one of the above

						symptoms accompanied by sore throat and nasal discharge within 5 days, fever without other cause, increased cough and an increase in respiratory rate or heart rate 20% above baseline values
Jackson (2021)[30]	USA	Prospective, multicentre	Secondary care	Influenza season, November 2018-April 2019	Incidence	Medically attended ARI: ARI and Cough illness for less than 8 days
Jain (2015)[31]	USA	Prospective, multicentre	Secondary care	January 2010-June 2012	Incidence	Acute infection: fever or chills, documented fever or hypothermia, leukocytosis or leukopenia, or new altered mental status<br>ARI: new cough or sputum production, chest pain, dyspnea, tachypnea, abnormal lung examination, or respiratory failure<br>Radiographic evidence of pneumonia: the presence of consolidation, other infiltrate, or pleural effusion
Jeannoel (2019)[32]	France	Retrospective, single centre	Secondary care	3 winter seasons (October to April), 2013-2016	Proportion	Respiratory symptoms
Johnstone (2008)[33]	Canada	Prospective, multicentre	Secondary care	January 2004-January 2006	Proportion	CAP: acute LRTI with two or more of cough, productive cough, fever, chills, dyspnea, pleuritic chest pain, crackles, and bronchial breathing plus an opacity or inflltrate seen on a chest radiograph that was interpreted as pneumonia by the physician
Johnstone (2014)[34]	Canada	Prospective, multicentre	Nursing homes	2009-2011	Proportion	Respiratory symptoms: new symptoms of fever ≥ 38°C, worsening cough, nasal congestion, sore throat, headache, sinus problems, muscle aches, fatigue, earache or infection, chills, not otherwise explained by an alternative
Juretschko (2017)[35]	USA	Prospective, multicentre	Secondary care	Influenza seasons, 2015-2016	Prevalence	Suspected RTI
Korsten (2021)[36]	Belgium, UK, Netherlan ds	Prospective, multicentre	Community and primary care	RSV seasons (October-May), 2017-2019	Proportion	ARTI: one or more of nasal congestion or discharge, cough, wheezing, or shortness of breath
Leli (2021)[37]	Italy	Retrospective, single centre	Secondary care	January 2016-June 2020	Prevalence	Symptoms of respiratory infection
Loubet (2017)[38]	France	Prospective, multicentre	Secondary care	2012-2015	Prevalence	ILI: at least one systemic symptom, fever ≥38C, headache, myalgia or malaise, and at least respiratory symptoms, cough, sore throat or dyspnoea for at least 24 hours with symptom onset <7 days before sampling

Louie (2005)[39]	USA	Prospective, single centre	Secondary care	January-March 2002	Prevalence	ARI: new illness within the past 3 weeks with cough, sinus pain, congestion, sore throat, or fever
Malosh (2017)[40]	USA	Prospective, multicentre	Secondary care	2 influenza seasons, November 2014-March 2015, November 2015-April 2016	Proportion	ARI
Marcos (2006)[41]	Spain	Prospective, single centre	Secondary care	January 2003-March 2004	Incidence	CAP: presence of a new infiltrate on chest radiography with clinical symptoms suggestive of LRTI and no alternative diagnosis in a patient not admitted to hospital within the previous month and no alternative diagnosis was established during follow-up
McClure (2014)[42]	USA	Prospective, multicentre	Primary and secondary care	4 influenza seasons, 2006-2010	Incidence	ARI: symptoms of fever/feverishness, chills, or cough
Pellegrinelli (2020)[43]	Italy	Retrospective, multicentre	Primary care	4 winter/influenza seasons, 2014-2018	Prevalence	ILI: abrupt onset of fever >38°C or feverishness, one or more respiratory symptoms, cough, sore throat and/or shortness of breath and one or more systemic symptoms, myalgia, headache and/or malaise
Poole (2020)[44]	UK	Prospective, multicentre	Secondary and tertiary care	March-May, 2015-2020	Proportion	ARI or suspected viral respiratory tract infection, recruited within 24 hours of presentation to hospital
Prasad (2020)[45]	New Zealand	Retrospective, multicentre	Secondary care	Winter seasons (weeks 18-39), 2012–2015	Incidence	ARI (SARI or non-SARI)<br>SARI: cough and measured or reported fever within the last 7 days (2012) and within 10 days (from 2013)
Prasad (2021)[46]	New Zealand	Prospective, multicentre	Secondary care	Winter seasons, 2012-2015	Prevalence	SARI: cough and measured or reported fever within the last 7 days (2012) and the last 10 days (2013–2015)
Price (2019)[47]	Australia	Prospective, multicentre	Infectious Diseases Reference Laboratory	May 2002-December 2017	Proportion	Testing deemed clinically relevant
Reckziegel (2020)[48]	Germany	Prospective	NR	September 2009-December 2012	Prevalence	RTI: common cold, cough with or without sputum, dyspnea, and fever
Saez-Lopez (2019)[49]	Portugal	Retrospective, multicentre	Primary and secondary care	Winter seasons (week 40-week 20), 2010-2018	Proportion	ILI: sudden onset of at least one respiratory symptom, cough, sore throat, shortness of breath and at least one systemic symptom, fever or feverishness, malaise, headache or myalgia

Saez-Lopez (2019)[50]	Portugal	Retrospective, multicentre	Primary and secondary care	4 Influenza seasons (week 37-week 24), 2014-2018	Prevalence	ILI<br>Respiratory infection (2017-2018)
Self (2016)[51]	USA	Prospective, multicentre	Primary care	February 2011-June 2012	Prevalence	Asymptomatic adults: absence of fever, cough, sore throat, wheeze, shortness of breath, rhinorrhea, ear pain, and vomiting for past 14 days and CAP patients: acute infection, ARI, and chest imaging showing consolidation, infiltrate or pleural effusion
Shorr (2018)[52]	USA	Prospective, single centre	Community and secondary care	January-December 2016	Prevalence	Respiratory failure requiring MV in pneumonia patients
Smithgall (2020)[53]	USA	Prospective, multicentre	Primary care and community	January 2013-December 2015	Proportion	Hospitalised population: respiratory symptoms<br>Community: ARI ≥2 symptoms of fever/feverishness, cough, sore throat, runny nose/nasal congestion or body aches or ILI
Stamm (2021)[54]	Germany	Retrospective, single centre	Tertiary care	2018-2021	Incidence	None specified; sample was patients who underwent testing for viral respiratory disease
Subissi (2020)[55]	Belgium	Prospective, multicentre	Secondary care	Influenza season (week 40-week 20), 2018-2019	Proportion	SARI: ARI with fever ≥38°C (or history of fever reported by patient) and cough or dyspnoea, with onset of symptoms within the past 10 days, requiring hospitalisation (minimum overnight)
Subissi (2021)[56]	Belgium	Prospective, multicentre	Primary and secondary care	4 Influenza seasons (week 40-week 20), 2015-2019	Incidence<br>Proportion	SARI: ARI with fever of ≥38°C and with cough and/or dyspnoea within previous 10 days and overnight hospital stay<br>ILI: fever and cough or dyspnoea
Sumino (2010)[57]	USA	Prospective, single centre	Secondary care	October 2005-October 2006	Prevalence	ARI
Sundaram (2014)[58]	USA	Prospective, multicentre	Primary and secondary care	2004-2005 to 2009-2010 (January-March, delayed to March-April in 2005–2006)	Proportion	Medically attend ARI (2004 – 2007) and subsequently respiratory illness and at least 1 symptom: feverishness, chills, or cough
Tanner (2012)[59]	UK	Retrospective, single centre	Public health laboratory	Winter season, 2009-2010	Proportion	ARI
Tramuto (2021)[60]	Italy	Retrospective, multicentre	Primary and secondary care	5 winter seasons (October-April), 2015-2020	Prevalence	ILI outpatients and SARI inpatients
Tsagarakis (2018)[61]	Greece	Retrospective, multicentre	Laboratories and tertiary care	2015-2016	Prevalence	Symptomatic RTI

Ursic (2016)[62]	Slovenia	Prospective, single centre	Nursing home	December 2011-May 2012	Incidence	ARI
Van Beek (2017)[63]	Netherlands	Prospective, multicentre	Community	2 influenza seasons, December 2011-April 2012, October 2012- May 2013	Incidence	ILI: fever ≥37.8°C with at least 1 other symptom of headache, myalgia, sore throat, coughing, rhinitis, or chest pain
Varghese (2018)[64]	Australia	Retrospective, multicentre	Primary care	January 2010-December 2013	Proportion	ILI: cough, fever and fatigue
Vos (2018)[65]	Netherlands	Prospective, multicentre	Primary and secondary care	12 viral respiratory seasons, 2005 (week 30)-2017 (week 29)	Incidence	ILI or ARI
Walsh (2004)[66]	USA	Prospective, single centre	Community and secondary care	2 winter seasons (November-April), 1999-2001	Proportion	High-risk adults: symptomatic congestive heart failure (CHF): a physician's diagnosis or an ejection fraction <35% or symptomatic pulmonary disease, primarily COPD<br>Hospitalised group: acute respiratory symptoms or diagnosis of underlying CHF or pulmonary disease<br>Healthy elderly persons: no disabling underlying illnesses
Wansaula (2016)[67]	USA	Prospective, multicentre	Secondary care	2010(week 40)-2014(week 39)	Proportion	SARI requiring hospitalisation: temperature ≥37.8°C or subjective fever or chills, in addition to cough, sore throat, or shortness of breath
Weinberger (2018)[68]	Germany	Prospective, multicentre	Primary care	October 2001-December 2004; October 2002-December 2004	Proportion	Long-lasting cough: coughed for 7 days or more
Widmer (2012)[69]	USA	Prospective, multicentre	Secondary care	Influenza seasons (November-April), 2006-2007, 2007-2008, 2008–2009	Proportion	Hospitalised with any respiratory symptoms: cough, nasal congestion, coryza, dyspnea, or wheezing) or non-localising fever
Widmer (2014)[70]	USA	Prospective, multicentre	Secondary care	May 2009 - April 2010	Proportion	Any respiratory symptoms or non-localising fever beginning within 7 days before presentation
Zambon (2001)[71]	UK	Prospective, multicentre	Primary care	3 winter seasons, 1995-1998	Proportion	ILI: symptoms of fever, cough and respiratory tract-illness

Abbreviations: ARI, acute respiratory illness; RSV, respiratory syncytial virus; ILI, influenza-like illness; SARI, severe acute respiratory illness; RTI, respiratory tract infection; COPD, chronic obstructive pulmonary disease; AECOPD, acute exacerbation of chronic obstructive pulmonary disease; LRTI, lower respiratory tract infection; CAP, community-acquired pneumonia; CCU, critical care unit; MV, mechanical ventilation; ARTI, acute respiratory tract infection

S4 Table. Population characteristics of included studies.

Author (year), country	Sample size (n)	Mean (SD) age [years]	Median (range) age [years]	Age groups evaluated (%) [years]*	Comorbidity required for inclusion
Belongia (2018), USA[1]	2,257	NR	NR	≥ 60 (100)	None
Bozio (2018), USA[2]	2,259	NR	57.0 (46.0-71.0)	> 18 (100)	None
Branche (2014), USA[3]	965	NR	NR	>21 (100)	None
Branche (2021), USA[4]	10,078	NR	NR	18–49 (14)<br>50-64 (27)<br>≥ 65 (59)<br>65-70 (21)<br>75-84 (20)<br>≥ 85 (18)	None
Brittain-Long (2010), Sweden[5]	Acute RTI patients:209	NR	NR	≥ 18 (100)	None
	Control group: 100	NR	NR	≥ 18 (100)	None
Camargo (2008), USA[6]	76	72.0 (NR)	NR	≥ 50 (100)	COPD
Carrat (2006), France[7]	122	69.0 (NR)	NR	NR	Chronic cardiac or pulmonary disorders
Charles (2008), Australia[8]	885	NR	NR	> 18 (100)	None
Chasqueira (2018), Portugal[9]	188	Men: 83 (9.2)<br>Women: 85 (7.2)	NR	NR	None
Ciotti (2020), Italy[10]	537	NR	NR	18-45 (NR)<br>46-64 (NR)<br>≥ 65 (NR)	None
Civljak (2019), Croatia[11]	182	NR	66.0 (19.0 - 94.0)	≥ 18 (100)	None
Creer (2005), UK[12]	LRTI patients: 80	49.9 (19.7)	NR (18.0–90.0)	≥ 18 (100)	None
	Control group: 49	49.7 (17.3)	NR (22.0-83.0)	≥ 18 (100)	None
De Francesco (2021), Italy[13]	Pre-pandemic: 10,121	65.1 (NR)	NR	18-44 (13.2)<br>56-64 (29.7)<br>65-79 (35.1)<br>≥80 (21.6)	None
	During pandemic: 2,362	63.5 (NR)	NR	18-44 (11.0)<br>56-64 (39.7)<br>65-79 (35.4)<br>≥80 (13.6)	None

De Serres (2009), Canada[14]	108	NR	NR	50–59 (14.0)<br>60–69 (29.0)<br>≥ 70 (57.0)	COPD
Descamps (2021), France[15]	1,428	68.0	NR (19.0 - 98.0)	<65 (NR)<br>≥65 (NR)	None
Diaz-Decaro (2018), USA[16]	52	NR	NR	<65 (22.7)<br>65-85 (46.0)<br>85+ (31.3)	None
Dimopoulos (2012), Greece[17]	200	69.7 (9.1)	NR	≥ 18 (100)	COPD/AECOPD
Falsey (2005), USA[18]	Healthy elderly adults: 608	75.0 (6.0)	NR	≥ 65 (100)	None
	High risk adults: 540	70.0 (11.0)	NR	≥ 21 (100)	Congestive heart failure/ chronic pulmonary disease
Falsey (2006), USA[19]	Respiratory cases: 146	42 (14)	NR	≥18 (100)	None
	Control subjects: 158	44 (15)	NR	≥ 18 (100)	None
Falsey (2008), USA[20]	382	84.9 (NR)	NR (65–102)	≥65 (100)	None
Falsey (2014), Multi-country[21]	553	72.8 (5.5)	NR	65-69 (32.2)<br>70-74 (32.7)<br>75-79 (22.4)<br>≥ 80 (12.7)	None
Galli (2020), Italy[22]	Self-sampled: 132	NR	38.8 (IQR: 26.5)	≥ 18 (100)	None
	GP sampled: 132	NR	43.4 (IQR: 17.9)	≥ 18 (100)	None
Gaymard (2018), France[23]	20,359	NR	NR	> 20 - ≤ 65 (25.0)<br>> 65 (21.0)	None
Gimferrer (2019), Spain[24]	31,692	NR	NR	> 64 (100)	None
Glezen (2000), USA[25]	1029	NR	NR	18-44 (28.8)<br>45-64 (45.6)<br>≥65 (25.6)	None/chronic underlying conditions
Hall (2001), USA[26]	2,960	30.0 (NR)	NR	18-60 (100)	None
Hause (2019), USA[27]	77	NR	NR	>18 (100)	None
Hirve (2020), Multi-country[28]	22,221	NR	NR	18-64 (NR)<br>≥ 65 (NR)	None
Hutchinson (2007), Australia[29]	148	72.0 (NR)	NR (49.0-85.0)	49-85 (100)	COPD

Jackson (2021), USA[30]	2,767	NR	NR	18-30 (12.5)<br>31-49 (23.2)<br>50-64 (20.5)<br>65+ (18.8)	None
Jain (2015), USA[31]	2,320	NR	57.0 (46.0-71.0)	18-49 (30.0)<br>50-64 (34.0)<br>65-79 (22.0)<br>≥ 80 (14.0)	None
Jeannoel (2019), France[32]	14,792	NR	NR	≥18 (100)	None
Johnstone (2008), Canada[33]	193	NR	71.0 (58.0 - 80.0)	≥ 18 (NR)	None
Johnstone (2014), Canada[34]	269	NR	NR	≥ 65 (NR)	None
Juretschko (2017), USA[35]	2,479	NR	NR	21-65 (34.1)<br>> 65 (29.5)	None
Korsten (2021), Belgium, UK, Netherlands[36]	1,040	NR	75.0 (60.0-100.0)	≥ 60 (100)<br>>75 (54)	None
Leli (2021), Italy[37]	572	NR	≥ 18 - < 65: 52 (43-57)<br>≥ 65: 73(70-79)	≥ 18 - < 65 (35.0)<br>≥ 65 (30.6)	None
Loubet (2017), France[38]	1,452	NR	70 .0 (54.0 – 82.0)	≥ 18 (100)	None
Louie (2005), USA[39]	266	38.7 (15.1)	38.0 (28.0-44.0)	≥ 18 (100)	None
Malosh (2017), USA[40]	1,259	NR	NR	18-49 (29.5)<br>50-64 (36.6)<br>≥65 (33.8)	None
Marcos (2006), Spain[41]	198	NR	70.0 (49.0-79.0)	Adults	None
McClure (2014), USA[42]	20,453	63.7 (12.1)	60.3 (NR)	50–59 (48.7)<br>60–69 (21.9)<br>≥70 (29.4)	None
Pellegrinelli (2020), Italy[43]	1,047	NR	NR	46-65 (23.8)<br>> 65 (11.9)	None
Poole (2020), UK[44]	856	NR	NR	≥ 18 (100)	None
Prasad (2020), New Zealand[45]	4,600	NR	NR	18–49 (NR)<br>50–64 (NR)<br>65-80 (NR)<br>≥ 80 (NR)	None

Prasad (2021), New Zealand[46]	3,875	NR	NR	18-49 (35.3)<br>50-64 (29.1)<br>65-80 (35.6)	Chronic medical condition
Price (2019), Australia[47]	33,652	NR	45.3 (NR)	20-64 (51.3)<br>65 (23.1)	None
Reckziegel (2020), Germany (unclear)[48]	Immunocompromised: 225	NR	46.2 (NR)	Adults (n = 188)	Immunocompromised
	Immunocompetent: 266	NR	57.2 (NR)	Adults (n = 167)	None
Saez-Lopez (2019), Portugal[49]	7,085	NR	NR	≥ 65 (n = 951)	None
Saez-Lopez (2019), Portugal[50]	20,670	NR	NR	45-64 (n = 3,830)<br>≥ 65 (n = 5,099)	None
Self (2016), USA[51]	Asymptomatic adults: 759	NR	54.0 (41.0–65.0)	≥ 18 (n = 238)	None
	CAP adults: 1,024	NR	59.0 (50.0–73.0)	≥ 18 (n = 192)	None
Shorr (2018), USA[52]	364	58.2 (15.2)	NR	≥ 18 (100)	None
Smithgall (2020), USA[53]	Hospitalised adults: 40,461 tests	NR	NR	≥ 18 (NR)	None
	Community adults: 1,805 tests	NR	NR	≥ 18 (NR)	None
Stamm (2021), Germany[54]	14,946	NR	NR	Adults (100)	None
Subissi (2020), Belgium[55]	508	NR	NR	≥ 65 (25.0)	None
Subissi (2021), Belgium[56]	ILI surveillance: 1,791	NR	NR	≥ 65 (n = 124)	None
	SARI surveillance: 4,774	NR	NR	≥ 65 (n = 2,105)	None
Sumino (2010)[57]	283	55 (15)	NR	Adults (100)	None
Sundaram (2014), USA[58]	2,225	64.3 (10.7)	NR	50-64 (55.4)<br>65-79 (33.7)<br>≥ 80 (10.9)	None†
Tanner (2012), UK[59]	4,821	NR	NR	25-44 (NR)<br>45-64 (NR)<br>65+ (NR)	None
Tramuto (2021), Italy[60]	9,584	NR	10.0 (IQR: 41.0)	19–34 (7.5)<br>35–49 (9.3)<br>50–64 (11.1)<br>≥ 65 (11.0)	None
Tsagarakis (2018), Greece[61]	656	NR	7.0 (0.1–92.0)	≥ 18 - < 45 (NR)<br>≥ 45 - < 65 (NR)<br>≥ 65 (NR)	None

Ursic (2016), Slovenia[62]	Nursing home residents: 90	NR	84.0 (79.8–88.8)	NR	None
	Nursing home staff: 42	NR	38.2 (33.5–46.1)	NR	None
Van Beek (2017), Netherlands[63]	2011-2012 ILI cases: 141	68.8 (NR)	NR	≥ 60 (100)	None
	2012-2013 ILI cases: 260	70.1 (NR)	NR	≥ 60 (100)	None
	2012-2013 Controls: 340	73.8 (NR)	NR	≥ 60 (100)	None
Varghese (2018), Australia[64]	5,031	NR	NR	20-49 (65.2)<br>50-64 (22.6)<br>≥ 65 (12.3)	None
Vos (2018), Netherlands[65]	13,577	NR	NR	45–65 (NR)<br>> 65–75 (NR)<br>> 75 (NR)	None
Walsh (2004), USA[66]	1999-2000 healthy elderly adults: 216	NR	NR	≥65 (100)	NR
	2000-2001 healthy elderly adults: 289	NR	NR	≥65 (100)	NR
	1999-2000 high risk adults: 204	NR	NR	≥21 (100)	Symptomatic congestive heart failure or symptomatic pulmonary disease
	2000-2001 high risk adults: 265	NR	NR	≥21 (100)	Symptomatic congestive heart failure or symptomatic pulmonary disease
	1999-2000 hospitalised adults: 315	NR	NR	≥65 (100)	NR
	2000-2001 hospitalised adults: 310	NR	NR	≥65 (100)	NR
Wansaula (2016), USA[67]	332	NR	63.0 (0.0-97.0)	25-49 (20.0)<br>50-64 (24.0)<br>≥ 65 (47.0)	None
Weinberger (2018), Germany[68]	975	NR	50.2 (18.0–87.8)	≥ 18 (100)	None
Widmer (2012), USA[69]	508	NR	66.0 (58.0-78.0)	50-64 (44.5)<br>≥ 65 (55.5)	None
Widmer (2014), USA[70]	1,248	NR	51.1 (NR)	18-49 (48.4)<br>≥ 50 (51.6)	None

Zambon (2001), UK[71]	2,226	NR	NR	45-65 (NR)<br>≥ 65 (NR)	None

Abbreviations: AECOPD, acute exacerbation of chronic obstructive pulmonary disease; CAP, community acquired pneumonia; COPD, chronic obstructive pulmonary disease; IQR, interquartile range; NR, not reported; SD, standard deviation * Children or adolescents were also evaluated in some studies †During the 2004-2005 season, patients aged 50-64 were eligible if they had at least 1 high-risk chronic medical condition.

S1 File. PRISMA 2009 Checklist.

Section/topic	#	Checklist item	Reported on page #
**TITLE**			
Title	1	Identify the report as a systematic review, meta-analysis, or both.	N.A. study design reported in page 5
**ABSTRACT**			
Structured summary	2	Provide a structured summary including, as applicable: background; objectives; data sources; study eligibility criteria, participants, and interventions; study appraisal and synthesis methods; results; limitations; conclusions and implications of key findings; systematic review registration number.	Page 2-3
**INTRODUCTION**			
Rationale	3	Describe the rationale for the review in the context of what is already known.	Page 3-4
Objectives	4	Provide an explicit statement of questions being addressed with reference to participants, interventions, comparisons, outcomes, and study design (PICOS).	Page 4-5
**METHODS**			
Protocol and registration	5	Indicate if a review protocol exists, if and where it can be accessed (e.g., Web address), and, if available, provide registration information including registration number.	Page 5
Eligibility criteria	6	Specify study characteristics (e.g., PICOS, length of follow-up) and report characteristics (e.g., years considered, language, publication status) used as criteria for eligibility, giving rationale.	Page 5-6
Information sources	7	Describe all information sources (e.g., databases with dates of coverage, contact with study authors to identify additional studies) in the search and date last searched.	Page 5
Search	8	Present full electronic search strategy for at least one database, including any limits used, such that it could be repeated.	Page 5 – S1 and S2 tables
Study selection	9	State the process for selecting studies (i.e., screening, eligibility, included in systematic review, and, if applicable, included in the meta-analysis).	Page 5-6
Data collection process	10	Describe method of data extraction from reports (e.g., piloted forms, independently, in duplicate) and any processes for obtaining and confirming data from investigators.	Page 5-6
Data items	11	List and define all variables for which data were sought (e.g., PICOS, funding sources) and any assumptions and simplifications made.	Page 6

Risk of bias in individual studies	12	Describe methods used for assessing risk of bias of individual studies (including specification of whether this was done at the study or outcome level), and how this information is to be used in any data synthesis.	N.A. justification provided in page 6
Summary measures	13	State the principal summary measures (e.g., risk ratio, difference in means).	N.A.
Synthesis of results	14	Describe the methods of handling data and combining results of studies, if done, including measures of consistency (e.g., I^2) for each meta-analysis.	N.A. qualitative synthesis only

**Section/topic**	**#**	**Checklist item**	**Reported on page #**
Risk of bias across studies	15	Specify any assessment of risk of bias that may affect the cumulative evidence (e.g., publication bias, selective reporting within studies).	N.A.
Additional analyses	16	Describe methods of additional analyses (e.g., sensitivity or subgroup analyses, meta-regression), if done, indicating which were pre-specified.	N.A.
**RESULTS**			
Study selection	17	Give numbers of studies screened, assessed for eligibility, and included in the review, with reasons for exclusions at each stage, ideally with a flow diagram.	Page 7
Study characteristics	18	For each study, present characteristics for which data were extracted (e.g., study size, PICOS, follow-up period) and provide the citations.	Page 7 – S1 Table
Risk of bias within studies	19	Present data on risk of bias of each study and, if available, any outcome level assessment (see item 12).	N.A.
Results of individual studies	20	For all outcomes considered (benefits or harms), present, for each study: (a) simple summary data for each intervention group (b) effect estimates and confidence intervals, ideally with a forest plot.	Table 2-3- Page 10-20
Synthesis of results	21	Present results of each meta-analysis done, including confidence intervals and measures of consistency.	N.A. synthesis of results provided in page 10-20
Risk of bias across studies	22	Present results of any assessment of risk of bias across studies (see Item 15).	N.A.
Additional analysis	23	Give results of additional analyses, if done (e.g., sensitivity or subgroup analyses, meta-regression [see Item 16]).	Table 3
**DISCUSSION**			
Summary of evidence	24	Summarize the main findings including the strength of evidence for each main outcome; consider their relevance to key groups (e.g., healthcare providers, users, and policy makers).	Page 20 -22

Limitations	25	Discuss limitations at study and outcome level (e.g., risk of bias), and at review-level (e.g., incomplete retrieval of identified research, reporting bias).	Page 22-24
Conclusions	26	Provide a general interpretation of the results in the context of other evidence, and implications for future research.	Page 24-25
**FUNDING**			
Funding	27	Describe sources of funding for the systematic review and other support (e.g., supply of data); role of funders for the systematic review.	Provided separately

From: Moher D, Liberati A, Tetzlaff J, Altman DG, The PRISMA Group (2009). Preferred Reporting Items for Systematic Reviews and Meta-Analyses: The PRISMA Statement. PLoS Med 6(7): e1000097. doi:10.1371/journal.pmed1000097

For more information, visit: **www.prisma-statement.org**.

References

1. Belongia EA, King JP, Kieke BA, Pluta J, Al-Hilli A, Meece JK, et al. Clinical Features, Severity, and Incidence of RSV Illness During 12 Consecutive Seasons in a Community Cohort of Adults >=60 Years Old. Open forum infect. 2018;5(12):ofy316.
2. Bozio CH, Flanders WD, Finelli L, Bramley AM, Reed C, Gandhi NR, et al. Use of Multiple Imputation to Estimate the Proportion of Respiratory Virus Detections Among Patients Hospitalized With Community-Acquired Pneumonia. Open forum infect. 2018;5(4):ofy061.
3. Branche AR, Walsh EE, Formica MA, Falsey AR. Detection of respiratory viruses in sputum from adults by use of automated multiplex PCR. J Clin Microbiol. 2014;52(10):3590-6.
4. Branche AR, Saiman L, Walsh EE, Falsey AR, Sieling WD, Greendyke W, et al. Incidence of Respiratory Syncytial Virus Infection among Hospitalized Adults, 2017-2020. Clinical Infectious Diseases. 2021;09:09.
5. Brittain-Long R, Westin J, Olofsson S, Lindh M, Andersson LM. Prospective evaluation of a novel multiplex real-time PCR assay for detection of fifteen respiratory pathogens-duration of symptoms significantly affects detection rate. J Clin Virol. 2010;47(3):263-7.
6. Camargo CA, Jr., Ginde AA, Clark S, Cartwright CP, Falsey AR, Niewoehner DE. Viral pathogens in acute exacerbations of chronic obstructive pulmonary disease. Intern Emerg Med. 2008;3(4):355-9.
7. Carrat F, Leruez-Ville M, Tonnellier M, Baudel JL, Deshayes J, Meyer P, et al. A virologic survey of patients admitted to a critical care unit for acute cardiorespiratory failure. Intensive Care Med. 2006;32(1):156-9.
8. Charles PG, Whitby M, Fuller AJ, Stirling R, Wright AA, Korman TM, et al. The etiology of community-acquired pneumonia in Australia: why penicillin plus doxycycline or a macrolide is the most appropriate therapy. Clin Infect Dis. 2008;46(10):1513-21.
9. Chasqueira MJ, Paixao P, Rodrigues ML, Piedade C, Caires I, Palmeiro T, et al. Respiratory infections in elderly people: Viral role in a resident population of elderly care centers in Lisbon, winter 2013-2014. Int J Infect Dis. 2018;69:1-7.
10. Ciotti M, Maurici M, Santoro V, Coppola L, Sarmati L, De Carolis G, et al. Viruses of Respiratory Tract: an Observational Retrospective Study on Hospitalized Patients in Rome, Italy. Microorganisms. 2020;8(4):01.
11. Civljak R, Tot T, Falsey AR, Huljev E, Vranes J, Ljubin-Sternak S. Viral pathogens associated with acute respiratory illness in hospitalized adults and elderly from Zagreb, Croatia, 2016 to 2018. J Med Virol. 2019;91(7):1202-9.
12. Creer DD, Dilworth JP, Gillespie SH, Johnston AR, Johnston SL, Ling C, et al. Aetiological role of viral and bacterial infections in acute adult lower respiratory tract infection (LRTI) in primary care. Thorax. 2006;61(1):75-9.
13. De Francesco MA, Pollara C, Gargiulo F, Giacomelli M, Caruso A. Circulation of Respiratory Viruses in Hospitalized Adults before and during the COVID-19 Pandemic in Brescia, Italy: A Retrospective Study. Int J Environ Res Public Health. 2021;18(18):09.
14. De Serres G, Lampron N, La Forge J, Rouleau I, Bourbeau J, Weiss K, et al. Importance of viral and bacterial infections in chronic obstructive pulmonary disease exacerbations. J Clin Virol. 2009;46(2):129-33.
15. Descamps A, Lenzi N, Galtier F, Laine F, Lesieur Z, Vanhems P, et al. In-hospital and midterm out-hospital complications of adults hospitalised with respiratory syncytial virus infection in France, 2017-2019: an observational study. Eur Respir J. 2021;26:26.
16. Diaz-Decaro JD, Launer B, McKinnell JA, Singh R, Dutciuc TD, Green NM, et al. Bayesian evidence and epidemiological implications of environmental contamination from acute respiratory infection in long-term care facilities. Epidemiology & Infection. 2018;146(7):832-8.
17. Dimopoulos G, Lerikou M, Tsiodras S, Chranioti A, Perros E, Anagnostopoulou U, et al. Viral epidemiology of acute exacerbations of chronic obstructive pulmonary disease. Pulm Pharmacol Ther. 2012;25(1):12-8.

18. Falsey AR, Hennessey PA, Formica MA, Cox C, Walsh EE. Respiratory syncytial virus infection in elderly and high-risk adults. N Engl J Med. 2005;352(17):1749-59.
19. Falsey AR, Criddle MC, Walsh EE. Detection of respiratory syncytial virus and human metapneumovirus by reverse transcription polymerase chain reaction in adults with and without respiratory illness. J Clin Virol. 2006;35(1):46-50.
20. Falsey AR, Dallal GE, Formica MA, Andolina GG, Hamer DH, Leka LL, et al. Long-term care facilities: a cornucopia of viral pathogens. J Am Geriatr Soc. 2008;56(7):1281-5.
21. Falsey AR, McElhaney JE, Beran J, van Essen GA, Duval X, Esen M, et al. Respiratory syncytial virus and other respiratory viral infections in older adults with moderate to severe influenza-like illness. J Infect Dis. 2014;209(12):1873-81.
22. Galli C, Pellegrinelli L, Del Castillo G, Forni G, Gandolfi CE, Mosillo M, et al. Self-Sampling of Oropharyngeal Swabs Among Healthcare Workers for Molecular Detection of Respiratory Viruses: A Valuable Approach for Epidemiological Studies and Surveillance Programs. Front. 2020;8:511669.
23. Gaymard A, Bouscambert-Duchamp M, Pichon M, Frobert E, Vallee J, Lina B, et al. Genetic characterization of respiratory syncytial virus highlights a new BA genotype and emergence of the ON1 genotype in Lyon, France, between 2010 and 2014. Journal of Clinical Virology. 2018;102:12-8.
24. Gimferrer L, Vila J, Pinana M, Andres C, Rodrigo-Pendas JA, Peremiquel-Trillas P, et al. Virological surveillance of human respiratory syncytial virus A and B at a tertiary hospital in Catalonia (Spain) during five consecutive seasons (2013-2018). Future Microbiol. 2019;14:373-81.
25. Glezen WP, Greenberg SB, Atmar RL, Piedra PA, Couch RB. Impact of respiratory virus infections on persons with chronic underlying conditions. Jama. 2000;283(4):499-505.
26. Hall CB, Long CE, Schnabel KC. Respiratory syncytial virus infections in previously healthy working adults. Clin Infect Dis. 2001;33(6):792-6.
27. Hause AM, Avadhanula V, Maccato ML, Pinell PM, Bond N, Santarcangelo P, et al. Clinical characteristics and outcomes of respiratory syncytial virus infection in pregnant women. Vaccine. 2019;37(26):3464-71.
28. Hirve S, Crawford N, Palekar R, Zhang W, Group WRs. Clinical characteristics, predictors, and performance of case definition-Interim results from the WHO global respiratory syncytial virus surveillance pilot. Influenza other respi. 2020;14(6):647-57.
29. Hutchinson AF, Ghimire AK, Thompson MA, Black JF, Brand CA, Lowe AJ, et al. A community-based, time-matched, case-control study of respiratory viruses and exacerbations of COPD. Respir Med. 2007;101(12):2472-81.
30. Jackson ML, Starita L, Kiniry E, Phillips CH, Wellwood S, Cho S, et al. Incidence of Medically Attended Acute Respiratory Illnesses Due to Respiratory Viruses Across the Life Course During the 2018/19 Influenza Season. Clinical Infectious Diseases. 2021;73(5):802-7.
31. Jain S, Self WH, Wunderink RG, Fakhran S, Balk R, Bramley AM, et al. Community-Acquired Pneumonia Requiring Hospitalization among U.S. Adults. N Engl J Med. 2015;373(5):415-27.
32. Jeannoel M, Lina G, Rasigade JP, Lina B, Morfin F, Casalegno JS. Microorganisms associated with respiratory syncytial virus pneumonia in the adult population. European Journal of Clinical Microbiology & Infectious Diseases. 2019;38(1):157-60.
33. Johnstone J, Majumdar SR, Fox JD, Marrie TJ. Viral infection in adults hospitalized with community-acquired pneumonia: prevalence, pathogens, and presentation. Chest. 2008;134(6):1141-8.
34. Johnstone J, Parsons R, Botelho F, Millar J, McNeil S, Fulop T, et al. Immune biomarkers predictive of respiratory viral infection in elderly nursing home residents. PLoS One. 2014;9(9):e108481.
35. Juretschko S, Mahony J, Buller RS, Manji R, Dunbar S, Walker K, et al. Multicenter Clinical Evaluation of the Luminex Aries Flu A/B & RSV Assay for Pediatric and Adult Respiratory Tract Specimens. J Clin Microbiol. 2017;55(8):2431-8.

36. Korsten K, Adriaenssens N, Coenen S, Butler CC, Verheij TJM, Bont LJ, et al. World Health Organization Influenza-Like Illness Underestimates the Burden of Respiratory Syncytial Virus Infection in Community-Dwelling Older Adults. Journal of Infectious Diseases. 2021;14:14.
37. Leli C, Di Matteo L, Gotta F, Vay D, Piceghello A, Cornaglia E, et al. Prevalence of respiratory viruses by Multiplex PCR: a four-and-a-half year retrospective study in an Italian general hospital. Infez Med. 2021;29(1):94-101.
38. Loubet P, Lenzi N, Valette M, Foulongne V, Krivine A, Houhou N, et al. Clinical characteristics and outcome of respiratory syncytial virus infection among adults hospitalized with influenza-like illness in France. Clin Microbiol Infect. 2017;23(4):253-9.
39. Louie JK, Hacker JK, Gonzales R, Mark J, Maselli JH, Yagi S, et al. Characterization of viral agents causing acute respiratory infection in a San Francisco University Medical Center Clinic during the influenza season. Clin Infect Dis. 2005;41(6):822-8.
40. Malosh RE, Martin ET, Callear AP, Petrie JG, Lauring AS, Lamerato L, et al. Respiratory syncytial virus hospitalization in middle-aged and older adults. Journal of Clinical Virology. 2017;96:37-43.
41. Angeles Marcos M, Camps M, Pumarola T, Antonio Martinez J, Martinez E, Mensa J, et al. The role of viruses in the aetiology of community-acquired pneumonia in adults. Antivir Ther. 2006;11(3):351-9.
42. McClure DL, Kieke BA, Sundaram ME, Simpson MD, Meece JK, Sifakis F, et al. Seasonal incidence of medically attended respiratory syncytial virus infection in a community cohort of adults ≥50 years old. PLoS One. 2014;9(7):e102586.
43. Pellegrinelli L, Galli C, Bubba L, Cereda D, Anselmi G, Binda S, et al. Respiratory syncytial virus in influenza-like illness cases: Epidemiology and molecular analyses of four consecutive winter seasons (2014-2015/2017-2018) in Lombardy (Northern Italy). J Med Virol. 2020;21:21.
44. Poole S, Brendish NJ, Clark TW. SARS-CoV-2 has displaced other seasonal respiratory viruses: Results from a prospective cohort study. J Infect. 2020;81(6):966-72.
45. Prasad N, Newbern EC, Trenholme AA, Thompson MG, McArthur C, Wong CA, et al. The health and economic burden of respiratory syncytial virus associated hospitalizations in adults. PLoS ONE [Electronic Resource]. 2020;15(6):e0234235.
46. Prasad N, Walker TA, Waite B, Wood T, Trenholme AA, Baker MG, et al. Respiratory Syncytial Virus-Associated Hospitalizations Among Adults With Chronic Medical Conditions. Clinical Infectious Diseases. 2021;73(1):e158-e63.
47. Price OH, Sullivan SG, Sutterby C, Druce J, Carville KS. Using routine testing data to understand circulation patterns of influenza A, respiratory syncytial virus and other respiratory viruses in Victoria, Australia. Epidemiology & Infection. 2019;147:e221.
48. Reckziegel M, Weber-Osel C, Egerer R, Gruhn B, Kubek F, Walther M, et al. Viruses and atypical bacteria in the respiratory tract of immunocompromised and immunocompetent patients with airway infection. European Journal of Clinical Microbiology & Infectious Diseases. 2020;39(8):1581-92.
49. Saez-Lopez E, Pechirra P, Costa I, Cristovao P, Conde P, Machado A, et al. Performance of surveillance case definitions for respiratory syncytial virus infections through the sentinel influenza surveillance system, Portugal, 2010 to 2018. Euro Surveill. 2019;24(45).
50. Saez-Lopez E, Cristovao P, Costa I, Pechirra P, Conde P, Guiomar R, et al. Epidemiology and genetic variability of respiratory syncytial virus in Portugal, 2014-2018. Journal of Clinical Virology. 2019;121:104200.
51. Self WH, Williams DJ, Zhu Y, Ampofo K, Pavia AT, Chappell JD, et al. Respiratory Viral Detection in Children and Adults: Comparing Asymptomatic Controls and Patients With Community-Acquired Pneumonia. J Infect Dis. 2016;213(4):584-91.
52. Shorr AF, Fisher K, Micek ST, Kollef MH. The Burden of Viruses in Pneumonia Associated With Acute Respiratory Failure: An Underappreciated Issue. Chest. 2018;154(1):84-90.

53. Smithgall M, Maykowski P, Zachariah P, Oberhardt M, Vargas CY, Reed C, et al. Epidemiology, clinical features, and resource utilization associated with respiratory syncytial virus in the community and hospital. Influenza other respi. 2020;14(3):247-56.
54. Stamm P, Sagoschen I, Weise K, Plachter B, Munzel T, Gori T, et al. Influenza and RSV incidence during COVID-19 pandemic-an observational study from in-hospital point-of-care testing. Med Microbiol Immunol (Berl). 2021;210(5-6):277-82.
55. Subissi L, Bossuyt N, Reynders M, Gerard M, Dauby N, Bourgeois M, et al. Capturing respiratory syncytial virus season in Belgium using the influenza severe acute respiratory infection surveillance network, season 2018/19. Euro Surveill. 2020;25(39):10.
56. Subissi L, Bossuyt N, Reynders M, Gerard M, Dauby N, Lacor P, et al. Spotlight influenza: Extending influenza surveillance to detect non-influenza respiratory viruses of public health relevance: analysis of surveillance data, Belgium, 2015 to 2019. Euro Surveill. 2021;26(38):09.
57. Sumino KC, Walter MJ, Mikols CL, Thompson SA, Gaudreault-Keener M, Arens MQ, et al. Detection of respiratory viruses and the associated chemokine responses in serious acute respiratory illness. Thorax. 2010;65(7):639-44.
58. Sundaram ME, Meece JK, Sifakis F, Gasser RA, Jr., Belongia EA. Medically attended respiratory syncytial virus infections in adults aged ≥ 50 years: clinical characteristics and outcomes. Clin Infect Dis. 2014;58(3):342-9.
59. Tanner H, Boxall E, Osman H. Respiratory viral infections during the 2009-2010 winter season in Central England, UK: incidence and patterns of multiple virus co-infections. Eur J Clin Microbiol Infect Dis. 2012;31(11):3001-6.
60. Tramuto F, Maida CM, Di Naro D, Randazzo G, Vitale F, Restivo V, et al. Respiratory Syncytial Virus: New Challenges for Molecular Epidemiology Surveillance and Vaccination Strategy in Patients with ILI/SARI. 2021;9(11):16.
61. Tsagarakis NJ, Sideri A, Makridis P, Triantafyllou A, Stamoulakatou A, Papadogeorgaki E. Age-related prevalence of common upper respiratory pathogens, based on the application of the FilmArray Respiratory panel in a tertiary hospital in Greece. Medicine (Baltimore). 2018;97(22):e10903.
62. Uršič T, Miksić NG, Lusa L, Strle F, Petrovec M. Viral respiratory infections in a nursing home: a six-month prospective study. BMC Infect Dis. 2016;16(1):637.
63. van Beek J, Veenhoven RH, Bruin JP, van Boxtel RAJ, de Lange MMA, Meijer A, et al. Influenza-like Illness Incidence Is Not Reduced by Influenza Vaccination in a Cohort of Older Adults, Despite Effectively Reducing Laboratory-Confirmed Influenza Virus Infections. Journal of Infectious Diseases. 2017;216(4):415-24.
64. Varghese BM, Dent E, Chilver M, Cameron S, Stocks NP. Epidemiology of viral respiratory infections in Australian working-age adults (20-64 years): 2010-2013. Epidemiology & Infection. 2018;146(5):619-26.
65. Vos LM, Teirlinck AC, Lozano JE, Vega T, Donker GA, Hoepelman AI, et al. Use of the moving epidemic method (MEM) to assess national surveillance data for respiratory syncytial virus (RSV) in the Netherlands, 2005 to 2017. Euro Surveill. 2019;24(20).
66. Walsh EE, Peterson DR, Falsey AR. Risk factors for severe respiratory syncytial virus infection in elderly persons. J Infect Dis. 2004;189(2):233-8.
67. Wansaula Z, Olsen SJ, Casal MG, Golenko C, Erhart LM, Kammerer P, et al. Surveillance for severe acute respiratory infections in Southern Arizona, 2010-2014. Influenza Other Respir Viruses. 2016;10(3):161-9.
68. Weinberger R, Riffelmann M, Kennerknecht N, Hulse C, Littmann M, O'Brien J, et al. Long-lasting cough in an adult German population: incidence, symptoms, and related pathogens. European Journal of Clinical Microbiology & Infectious Diseases. 2018;37(4):665-72.
69. Widmer K, Zhu Y, Williams JV, Griffin MR, Edwards KM, Talbot HK. Rates of hospitalizations for respiratory syncytial virus, human metapneumovirus, and influenza virus in older adults. J Infect Dis. 2012;206(1):56-62.

70. Widmer K, Griffin MR, Zhu Y, Williams JV, Talbot HK. Respiratory syncytial virus- and human metapneumovirus-associated emergency department and hospital burden in adults. Influenza Other Respir Viruses. 2014;8(3):347-52.
71. Zambon MC, Stockton JD, Clewley JP, Fleming DM. Contribution of influenza and respiratory syncytial virus to community cases of influenza-like illness: an observational study. Lancet. 2001;358(9291):1410-6.
